# A method of active case detection to target reservoirs of asymptomatic malaria and gametocyte carriers in a rural area in Southern Province, Zambia

**DOI:** 10.1186/1475-2875-9-265

**Published:** 2010-10-04

**Authors:** Gillian H Stresman, Aniset Kamanga, Petros Moono, Harry Hamapumbu, Sungano Mharakurwa, Tamaki Kobayashi, William J Moss, Clive Shiff

**Affiliations:** 1 Department of Molecular Microbiology and Immunology, Johns Hopkins Bloomberg School of Public Health, Baltimore, MD, USA; 2 The Malaria Institute at Macha, P.O. Choma, Zambia; 3 Department of Epidemiology, Johns Hopkins Bloomberg School of Public Health, Baltimore, MD, USA

## Abstract

**Background:**

Asymptomatic reservoirs of malaria parasites are common yet are difficult to detect, posing a problem for malaria control. If control programmes focus on mosquito control and treatment of symptomatic individuals only, malaria can quickly resurge if interventions are scaled back. Foci of parasite populations must be identified and treated. Therefore, an active case detection system that facilitates detection of asymptomatic parasitaemia and gametocyte carriers was developed and tested in the Macha region in southern Zambia.

**Methods:**

Each week, nurses at participating rural health centres (RHC) communicated the number of rapid diagnostic test (RDT) positive malaria cases to a central research team. During the dry season when malaria transmission was lowest, the research team followed up each positive case reported by the RHC by a visit to the homestead. The coordinates of the location were obtained by GPS and all consenting residents completed a questionnaire and were screened for malaria using thick blood film, RDT, nested-PCR, and RT-PCR for asexual and sexual stage parasites. Persons who tested positive by RDT were treated with artemether/lumefantrine (Coartem^®^). Data were compared with a community-based study of randomly selected households to assess the prevalence of asymptomatic parasitaemia in the same localities in September 2009.

**Results:**

In total, 186 and 141 participants residing in 23 case and 24 control homesteads, respectively, were screened. In the case homesteads for which a control population was available (10 of the 23), household members of clinically diagnosed cases had a 8.0% prevalence of malaria using PCR compared to 0.7% PCR positive individuals in the control group (p = 0.006). The case and control groups had a gametocyte prevalence of 2.3% and 0%, respectively but the difference was not significant (p = 0.145).

**Conclusions:**

This pilot project showed that active case detection is feasible and can identify reservoirs of asymptomatic infection. A larger sample size, data over multiple low transmission seasons, and in areas with different transmission dynamics are needed to further validate this approach.

## Background

Asymptomatic malaria infections exist despite interventions directed at restricting transmission through mosquito control and treatment of symptomatic cases. In areas where *Plasmodium falciparum *is holoendemic, over half the population may be parasitaemic and show no obvious clinical symptoms [1]. The presence of asymptomatic malaria can also be seen to a lesser extent under unstable transmission conditions [2]. Unstable malaria is typically the result of seasonal influences. There is a period when mosquito populations are at a minimum and asymptomatic infections likely become a refuge for the parasite population and the source of new infections when mosquito populations expand [3,4]. These peaks and troughs of malaria infection offer an opportunity to target these reservoirs during the seasonal intercessions.

Among the asymptomatic cases are gametocyte carriers [2,4]. Asymptomatic infections can be associated with high levels of gametocytes [5], and likely serve as an important parasite reservoir [6]. If asymptomatic individuals can be identified and treated effectively with gametocytocidal drugs at a time when mosquito activities are minimal, it may be possible to reduce and eventually eliminate the parasite reservoir.

It is difficult to detect people who are parasitaemic but asymptomatic. If people are not experiencing symptoms, they will not actively seek testing for malaria. Even if they do seek care, levels of parasitaemia are often below that detected by blood slide examination [1]. Targeting these populations using appropriate technology, like rapid diagnostic tests (RDT), rather than resorting to molecular methods that are not feasible in most endemic areas in Africa would be helpful. Not only would such a system reduce the parasite reservoir but would enable local health personnel to implement such a programme that avoids testing or treating the entire population.

In areas with seasonal transmission, pockets of asymptomatic infection persist through the dry season and perpetuate disease transmission into the next season [3-5]. If a household has a member with malaria, it is more likely that there will be other infected persons clustered in that same unit [7]. Field experience and evidence from Zambia [8] formed the basis for an hypothesis that in an area where malaria control is implemented, symptomatic cases of malaria that appear during the low transmission season do not occur in isolation but represent a group of people sustaining asymptomatic infections. This paper presents the results of a pilot study conducted in the Southern Province of Zambia during the low transmission season that assessed the ability to detect local foci of asymptomatic infection by targeting members of homesteads of clinically-diagnosed cases of symptomatic *Plasmodium falciparum *malaria. RDTs were conducted at the identified homesteads and the prevalence of asymptomatic parasitaemia was measured by molecular methods in these targeted homesteads and compared to that found in randomly-selected control homesteads.

## Methods

**Figure 1:**
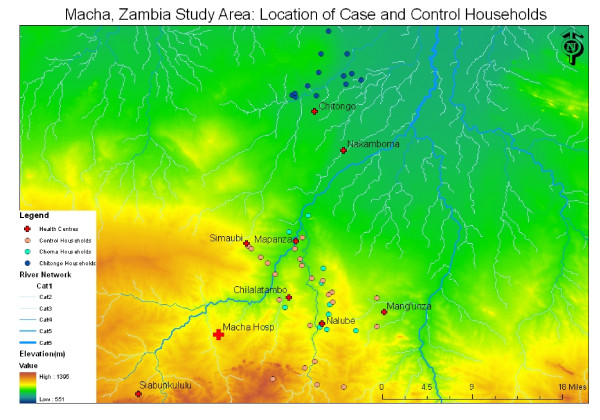
**Macha, Zambia Study Area: Location of Case and Control Homesteads**. The map shows the elevation and major river systems of the Macha study region as derived from a digital elevation model. The rural health centres in the area are shown (red cross). The active case detection system focused on the Nalube, Chilalantambo, Mapanza, and Chitongo health centres. The location of the homesteads included in the active case detection system in the Choma (pink) and Namwala (blue) districts and the randomly selected control homesteads (green) are also shown.

The study site consisted of a rural area covering about 7,000 km^2 ^in the Choma and Namwala districts in the Southern Province of Zambia (Figure 1). The majority of people live in homesteads, a group of buildings occupied by an extended family located in proximity to their farmland. Some homesteads are close together while others are isolated. This region experiences highly seasonal transmission of *P. falciparum*, with peak transmission occurring during the rainy season from November through May. The principal vector is *Anopheles arabiensis *and there are vector control measures in place. Insecticide-treated bed net (ITN) distribution covered the study area by June 2008. In 2007 the government began supplying rural health centres (RHC) with stocks of RDTs and requiring the use of these tests to diagnose malaria before treatment [9]. The first line of treatment for malaria in Zambia is artemether/lumefantrine combination therapy (Coartem^®^), with sulphadoxine-pyrimethamine (SP, Fansidar^®^) used for pregnant women.

A passive case detection system involving 13 RHCs in the Macha area has been in operation since 2008 and was described in detail [8]. Briefly, a staff member at each clinic sends a text message using their personal cell phone to a member of the research team every week with the number of cases of RDT confirmed malaria presenting at the RHC. The data are entered into a central database. From June to August 2009, the homesteads of all RDT confirmed cases of malaria captured by the passive case detection system were selected for the targeted screening process. The homestead where the index case resided was screened within two weeks of the case presenting at the RHC. This active case detection system was conducted at four RHCs in two different districts: Mapanza, Chilalantambo, and Nalube RHCs in Choma District, and Chitongo RHC in the Namwala District (Figure 1). These RHCs were identified because, except for one case, they were the only health centres participating in the passive case detection system that reported RDT confirmed cases of malaria from June to August 2009.

The control population participated in an ongoing community-based study of randomly selected homesteads to assess the prevalence of asymptomatic infections. Satellite imagery was used to identify and randomly select homesteads in the study area. Starting in 2007, individuals residing in these homesteads were screened for malaria. To approximate similar transmission dynamics in case and control homesteads, only data collected from homesteads during a similar time period were included. As no homesteads participating in the cross sectional study were screened between June and August 2009, data from September 2009 were used for comparison. No control population was available from Namwala District.

Screening of individuals residing in homesteads of clinically-diagnosed cases involved two or three visits by the research team. When an RDT confirmed case was identified at an RHC, the first visit was initiated. A field worker travelled by motorbike to the clinic and obtained the name of the case, located the homestead where the individual resided, recorded the location by handheld GPS, explained the study to the family, and obtained permission to return with the full study team for screening. The second visit occurred on the day and time agreed upon with the head of household, and was usually one to three days after the initial meeting and less than two weeks after the case initially presented to the RHC. The full screening took place on this visit. The study team obtained informed consent from all individuals who resided in the homestead for at least two weeks prior to the visit, and administered a short questionnaire. A blood specimen was obtained by a single finger prick and a thick blood film was prepared, an RDT was administered, and a Whatman 903 protein saver card was spotted. All RDT positive individuals were treated according to the Zambian Ministry of Health guidelines. Slides were stored in slide boxes and filter papers were placed in individual sealable plastic bags with desiccant and transported to the Macha Research Trust laboratory for analysis. Slides were stored at room temperature and protein saver cards at -20°C.

Blood samples were used to detect the presence of asexual and sexual stage malaria parasites using microscopy, as well as by nested PCR and RT-PCR. Two trained microscopists read the thick films. In the case of a discordant result, a third reading was done. Standard procedures for parasite counts were performed by microscopy within 48 hours of testing, and arrangements were made to visit and treat cases.

To detect subpatent cases, molecular methods were used to detect the asexual and sexual stages of the malaria parasite. Parasite DNA was extracted from the blood spotted on filter papers using the chelex method [10,11]. Extracted DNA was stored at -20°C. Nested PCR was conducted twice on each sample to increase the sensitivity; the first targeting the Plasmodium specific *cyt-b *target gene [12] and the second targeting the *P. falciparum *specific *msp-2 *gene [13] (Table 1). A sample was considered positive if either Nested-PCR showed a positive result. The *cyt-b *protocol was the same as that listed by Steenkeste *et al *[12], except that 6 μl of DNA template was used in both the primary and nested step. The second nested PCR was conducted on each sample with primers targeting the *P. falciparum *specific *msp-2 *gene as developed by Snounou *et al *[13] (table 1). Both the primary and nested PCR consisted of a 25 μL reaction with 10 × PCR buffer, 0.2 mM dNTP, 1.5 mM MgCl_2_, 0.26 μM of each primer, 1.25 units Taq DNA polymerase, and 4 μL of DNA template. Thermal cycler conditions consisted of 94°C for 5 minutes; 32 cycles of 94°C for 30 seconds, 50°C for 60 seconds, and 68°C for 120 seconds; and final extension at 72°C for 4 minutes. Samples were run on 1.5% agarose gel stained with ethidium bromide.

**Table 1:** Primer sequences for asexual P. falciparum DNA detection

Target: CYT-B (will detect all species of human *Plasmodium*)		
Primary (GCD)	F	5' CGG TCG CGT CCG GTA GCG TCT AAT GCC TAG ACG TAT TCC TGA TTA TCC AG 3'
	R	5' CGC ATC ACC TCT GGG CCG CGT GTT TGC TTG GGA GCT GTA ATC ATA ATG TG 3'

Nested (PLAS)	F	5' GAG AAT TAT GGA GTG GAT GGT G 3'
	R	5' TGG TAA TTG ACA TCC AAT CC 3'

Target: MSP-2 (will detect *P. falciparum *only)		

Primary	F	5' ATG AAG GTA ATT AAA ACA TTG TCT ATT ATA '3
(M20)	R	5' CTT TGT TAC CAT CGG TAC ATT CTT '3
Nested (IC)	F	5' AGA AGT ATG GCA GAA AGT AAK CCT YCT ACT 3'
	R	5' GAT TGT AAT TCG GGG GAT TCA GTT TGT TCG 3'

Nested RT-PCR was used to detect subpatent gametocytes targeting the gametocyte specific Pfs25 gene [14]. RNA extractions were conducted using a QIAGEN RNeasy Mini Kit^© ^following the manufacturers instructions. GE Illustra Ready-To-Go RT-PCR beads were used for the cDNA preparation. The primary step of the RT-PCR was conducted following the manufacturer's instructions using the primary primer sequences listed in Table 2. The nested step was conducted using the conventional PCR method as described by Mlambo *et al *[14]. Samples were run on 1.0% agarose gel stained with ethidium bromide.

**Table 2:** Primer sequences for P. falciparum gametocytes using RT-PCR

Target gene: Pfs25		
Primary	491(F)	5'-ATC GAT ATG AAT AAA CTT TAC AGT TTG TTT CT-3'
	492 (R)	5'-GAA TTC TTA CAT TAT AAA AAA GCA TAC TC-3'
	
Nested	25-1(F)	5'-TAA TGC GAA AGT TAC CGT GG-3'
	25-2 (R)	5'-TCC ATC AAC AGC TTT ACA GG-3'

The questionnaires, RDT, and lab results were double entered into an EpiInfo database. Data analysis was conducted using STATA 10.0. Fisher's exact test was used to compare proportions in the case and control groups. Clustering of samples was not taken into account in the statistical analysis due to small sample size.

## Results

As the case homesteads were selected from Choma and Namwala Districts, with a control population only available for Choma District, two separate analyses were conducted. The first was to determine if there were any significant differences between the two case districts and the second to assess differences between the case and control populations from Choma District.

Results in the populations screened at the case homesteads in Choma and Namwala Districts were similar. There were 87 people screened in 10 homesteads in Choma District, and 99 people in 13 homesteads in Namwala District. The age, mean number of people in each homestead and the sex distribution of the populations residing in the homesteads in each district showed no significant difference (Table 3). Both areas had similar proportions of the population positive for malaria, with 3% positive by RDT, 6.1% positive by nested-PCR, and 2% positive by RT-PCR in Namwala District and 2.3%, 8%, and 2.9% in Choma District, respectively (p-value 0.6, 0.8, and 0.6, respectively).

**Table 3:** Characteristics of the two case study populations.

	Choma	Namwala	**P-value (Fisher's Exact**)
Total Homesteads Screened	10	13	
Mean no. people screened	8.7	7.6	
per homestead	(median = 8; min = 3, max = 16)	(median = 6; min = 3, max = 19)	
N	87	99	
≤ 5 years old (%)	20 (23.0)	26 (26.3)	
> 5 years old (%)	67 (77.0)	73 (73.7)	
Mean age			
≤ 5 years old (range)	2.6 (0-5)	2.5 (0-5)	0.61
> 5 years old (range)	22.33 (6-77)	21.17 (6-69)	
Sex, n male (%)	44 (50.6)	42 (42.4)	0.26
≤ 5 years old	11 (55.0)	10 (38.5)	
> 5 years old	33 (49.0)	32 (43.8)	
Reported taking malaria	0	0	
meds in last 2 weeks (%)			
Self reported bed net use (%)	36/58 (62.1)	46/60 (76.7)	0.085
≤ 5 years old	10/18 (55.6)	13/15 (86.7)	0.07
> 5 years old	26/40 (65.0)	33/45 (73.3)	0.48
Reported ever IRS (%)	8/86 (9.3)	0/99 (0)	
			
Malaria Diagnosis			
RDT +ve (%)	2/87 (2.3)	3/98 (3.1)	0.56
≤ 5 years old	0/20 (0)	0/25 (0)	
> 5 years old	2/67 (3.0)	3/73 (4.1)	1.0
PCR +ve (%)	7/87 (8.0)	6/99 (6.1)	0.77
≤ 5 years old	3/20 (15)	0/26 (0)	0.08
> 5 years old	4/67 (6.0)	6/73 (8.2)	0.75
RT-PCR +ve (%)	2/87 (2.9)	2/99 (2.0)	0.64
≤ 5 years old	1/20 (5.0)	0/26 (0)	0.43
> 5 years old	1/67 (1.5)	2/73 (2.7)	1.0

The second analysis considered the case and control populations screened in Choma District. Active case detection identified 11 homesteads for screening between June and August 2009, with one refusing to participate. The control population consisted of 24 randomly selected homesteads in September 2009. In total, 87 individuals participated in the case and 141 in the control selected homesteads (Table 4). The mean size of homesteads differed significantly (p < 0.0001), with case households having a mean of 8.7 participants screened (median = 8, range 3 - 19) and controls having a mean of 5.9 participants (median = 5.5, range 1 - 13).

**Table 4:** Characteristics of the Choma district case and control study populations

	Case	Control
Total Homesteads Screened	10	24
Mean no. people screened per homestead	8.7	5.9
	(median = 8, min = 3, max = 16)	(median = 5.5, min = 1, max = 13)
N	87	141
≤ 5 years old (%)	20 (23.0)	33 (23.4)
> 5 years old (%)	67 (77.0)	108 (76.6)
Mean age		
≤ 5 years old (range)	2.6 (0-5)	2.73 (0-5)
> 5 years old (range)	22.33 (6-77)	25.53 (6-77)
Sex, n male (%)	44 (50.6)	69 (48.9)
≤ 5 years old	11 (55.0)	15 (45.4)
> 5 years old	33 (49.0)	54 (50.0)
Reported taking malaria meds in last 2 weeks n (%)	0	43/141 (30.5)
Self reported bed net use (%)	36/58 (62.1)	43/129 (33.0)
≤ 5 years old	10/18 (55.6)	35/113 (31.0)
> 5 years old	26/40 (65.0)	8/16 (50.0)
Reported ever IRS n (%)	8/86 (9.3)	0

The targeted active case detection system identified a higher proportion of participants who were positive for malaria than the randomly selected controls (Table 5). In the case population, 2.3% of individuals were RDT positive. All those diagnosed by RDT were older than five years of age, and in those over five years of age, the RDT positive prevalence was 3.6%. The control population had a prevalence of 0.7% of RDT positive individuals, with the only RDT positive individual younger than five years of age; however, the difference between cases and controls was not statistically significant (p = 0.56).

**Table 5:** Results of diagnostic measures for malaria in the pooled case and control study populations

	Case	Control	P-value (Fishers Exact)
RDT +ve (%)	2/87 (2.3)	1/141 (0.7)	0.56
≤ 5 years old	0/20 (0)	1/33 (3.0)	0.434
> 5 years old	2/67 (3.0)	0/108 (0)	0.07
PCR +ve (%)	7/87 (8.0)	1/141 (0.71)	0.006
≤ 5 years old	3/20 (15.0)	1/33 (3.0)	0.145
> 5 years old	4/67 (6.0)	0/108 (0)	0.02
RT-PCR +ve (%)	2/87 (2.3)	0/141 (0)	0.145
≤ 5 years old	1/20 (5.0)	0/33 (0)	0.195
> 5 years old	1/67 (1.5)	0/108 (0)	0.202

The targeted case detection system in Choma District found significantly more PCR positive (8.0%) individuals compared to 0.7% in the control population (p = 0.006). When stratified by age (younger or older than 5 years), parasitaemia by PCR was only significantly different between case (4/67) and control (0/108) homesteads among participants older than five years of age (p = 0.02). A gametocyte prevalence of 2.3% was found by active case detection and none were found in the control population. All indicators assessed were similar between the case and control groups except for the use of malaria medication, and self-reported bed net use. The population targeted for the active case detection had significantly fewer people reporting to have taken malaria medications within two weeks prior to the screening in the targeted homesteads (0%) compared to (30%) in the population in the control homesteads (p < 0.0001). In the homesteads identified through active case detection, 62% reported that they had used a bed net the night before whereas, in the control population, only 33% of participants reported bed net use Although there was more bed net use in the case population, which also reported more individuals positive for malaria, the majority of the positive diagnoses were found in those who did not report sleeping under a bed net. Only one of the seven participants positive for malaria by PCR and none of those who were gametocyte positive reported sleeping under a bed net.

## Discussion

Malaria in areas of unstable transmission usually follows seasonal patterns of transmission as mosquito populations fluctuate, with the prevalence of parasitaemia at a minimum in the cooler dry season. It is at this time when asymptomatic infections of malaria are critical, as this reservoir is likely responsible for sustaining the parasite population from one transmission season to the next [8]. Developing an active case detection system to identify these parasite refugia within asymptomatic reservoirs is critical to the development of elimination programmes. In this pilot study, RDT confirmed cases of malaria presenting to RHC in the low transmission season were shown to be indicators of household reservoirs of asymptomatic infections. Importantly, targeting residents in homesteads of clinical cases detected a significantly higher proportion of asymptomatic, PCR positive individuals than was detected in randomly selected control homesteads (Table 5). Although the presence of gametocytes was not significantly different between individuals in the targeted and control homesteads, a gametocyte prevalence of 2.3% was found in the case population and none in the control group. If the trends observed in the RDT, PCR, and RT-PCR results are valid and representative, targeted active case detection programme can identify a proportion of the asymptomatic reservoir. The findings are consistent with the observation that parasitaemia clusters and that there is an increased risk of infection in homesteads having an index case with malaria [7,15].

Parasite loads fluctuate [16] and some positive individuals may have been missed as homesteads were only screened once. Also, some of the parasite refugia will exist in homesteads not captured by this targeted active case detection system. It will be important to determine exactly what proportion of the asymptomatic reservoir is targeted through this system and if this proportion is sufficient to impact on its size and malaria transmission. Also, further studies including a larger sample size, data collection over multiple low transmission seasons, and exploring risk factors associated with homesteads comprising the asymptomatic reservoir are needed. It is also important to expand the study area to include a higher prevalence of malaria and different transmission dynamics to develop a programme that can be implemented by local governments and malaria control organizations.

In addition to the population residing in the targeted homesteads having a higher prevalence of asymptomatic infections, other relevant trends were also observed. Homesteads with clinically diagnosed case of malaria were larger than the randomly selected controls. Household characteristics, including population density and the number of children residing in the homestead, have been shown to be at risk for malaria [17,18]. The larger household size could be related to these characteristics and in fact be the reason for the higher prevalence of malaria, but the larger household size may also be a risk factor related to the presence of the reservoir. With a larger sample size and a focus on detecting risk factors of asymptomatic parasite populations, this association could be explored to determine if homestead size has a role in the presence of an asymptomatic reservoir.

Not accounting for bed net use or other factors, the proportion of asymptomatic malaria cases was similar between children and adults in the targeted homesteads. Children carry higher parasite and gametocyte loads than adults, with the parasite populations tapering off as the individual acquires immunity [19]. If low levels of *P. falciparum *parasites and gametocytes are present in the adult population at similar rates to children, then adults would also provide a competent reservoir capable of maintaining the transmission cycle. The extent of the distribution of asymptomatic malaria infections in both adults and children must be further explored so that interventions aimed at targeting the asymptomatic reservoir can be better informed.

If the goal is to reduce transmission, then asymptomatic reservoirs, particularly gametocytes, must be targeted using local health infrastructure. To sustain local reduction in malaria, it is important to identify these foci and design a system that is easily implemented by local health infrastructure. For active case detection to be effectively integrated into local health systems, the most effective way to identify and treat individuals using the local tools available must be determined. PCR is the most sensitive test for asymptomatic malaria; however, in the majority of malaria endemic areas using this molecular method as the main diagnostic tool is not cost or time effective. Using RDT results instead of PCR may be able to identify the asymptomatic populations, but this study did not have adequate power to detect a difference because of small sample size. If further studies indicate that it is indeed possible to use symptomatic cases of malaria infection in the low transmission season as an indicator of asymptomatic malaria infections, then the most effective way for the health centres to run sustainable programmes using the tools and infrastructure already in place must be determined.

## Conclusions

Previous efforts for malaria control are based on scaling up efforts during times of high transmission and focusing on vector control to reduce the probability of transmission. This project proposes an alternative approach to malaria control: targeting malaria during periods of low transmission when it is most vulnerable to elimination. This pilot project showed that a targeted active case detection system is possible in mesoendemic areas and can identify reservoirs of asymptomatic malaria infections. In this case in Zambia where anti-malaria interventions are under way, it may solve the strategic challenge of implementing active case detection. Moreover, these findings are not only important for designing systems of active case detection, but they push the field of malaria control towards a yet underexplored aspect of the malaria lifecycle: the asymptomatic reservoirs of malaria parasites in human populations.

## Conflicting interests

The authors declare that they have no competing interests.

## Ethical approval

The study was performed with the approval from the University of Zambia Research Ethics Committee 004-01-07, and the Johns Hopkins School of Public Health Ethical Review Board IRB number 00000229.

## Authors' contributions

GS was overall coordinator, managed the work and data collection, conducted the PCR assays, analysed the data, and wrote the paper; AK assisted in managing the project, implemented the SMS data collection system, conducted data collection, provided GIS support, and ran the RT-PCR assays; PM liaised with rural health centres, and was the senior field worker involved with data collection and entry; HH managed the collection of the control data; SM was overall coordinator and director of the lab in Zambia; TK is the overall research coordinator for study that provided the data for the control population; WM is the principal investigator for the study that provided data for the control population; CS set up the project, was the principal investigator, and obtained funding. All authors read and approved the manuscript.
